# Edibles in Negroland

**Published:** 1859-03

**Authors:** 


Edibles in Negroland.—In our notice of African edibles in the March number for 1858, we had followed the traveler, Dr. Barth, (in his southern journey,) from Tripoli, on the Mediterranean, as far as Tasawa, in about 13° 40′ north latitude. We shall pursue the same course now as we did then, in mentioning, for the most part in the author's own language, the different articles of food used by the natives of the several districts visited by Dr. Barth, as best illustrating their dietetic usages, rather than to give a formal list of all of the articles eaten by the inhabitants of Central Africa.

Near Tasawa, "the whole country had a most interesting and cheerful appearance, villages and corn-fields succeeding each other with only short intervals of thick underwood, which contributed to give richer variety to the whole landscape, while the ground was undulating, and might sometimes be called hilly." The travelers met a numerous herd of fine cattle, belonging to Gozenákko, returning to their pasture-grounds, after having been watered—the bulls all with the beautiful hump, and of fine, strong limbs, but of moderate size and with small horns. Scarcely had this moving picture passed before their eyes, when another interesting and characteristic procession succeeded—a long troop of men, all carrying on their heads large baskets, filled with the fruit of the góreba, (*Crucifera* or *Hyphaene Thebaica*,) commonly called the gingerbread-tree, which, in many of the northern districts of Negroland, furnishes a most important article of food, and certainly seasons many dishes very pleasantly. Farther on, the fields were enlivened with cattle grazing on the stubble.

At Gozenákko the attention of Barth and his companions was excited by the clamorous offering for sale of a various assortment of the delicacies of the country, by crowds of women from the village. During the whole evening a discordant chime was rung upon the words "nono," (sour milk,) "may," (butter,) "dodowa," (the vegetable paste mentioned in our last notice,) "kuka," the young leaves of the *Adansonia*, which are used for making an infusion with which meat, or the "tuwo," is eaten, and yarn da daria, (the sweet ground-nut, which, when roasted, is one of the greatest delicacies of the country.) "Tuwu," called "fufu" on the western coast, (the common paste or hasty-pudding made of millet, which forms the ordinary food of the natives,) was not offered for sale.

At a well on the road to Kaltena, which is a little below the 13th parallel of north latitude, the travelers were invited to purchase the common vegetables of the country, such as gowaza, or yams, dankah, or sweet potatoes, dodowa, or the vegetable cakes mentioned above, ground-nuts, beans, and sour milk. Near this spot they found, besides the fan-palm, the dumma and kana, and the immense monkeybread-tree, (*Adansonia*,) with its colossal (now, January 30th, leafless)

branches, from which the long heavy "kauchi" were hanging down on slender mouse-tail stalks. Here, too, was seen, for the first time, the kadena, or butter-tree, (*Bassia Parkii,*) in the neighborhood of the ayaba, or banana, and the gonda, or papaya. Now and then a herd of cattle were seen dispersed over the rich pasturage-grounds, all of white color, and the bulls provided with a large fat hump, or "tozo," hanging down on one side. A little farther on, we are told that all the cattle were of a white and all the goats of a coffee-brown color. Chemico-physiological reasons have been assigned for the white color of the polar bears and other animals in the arctic circle. What explanation will be offered for the prevalence of this same color among animals in the intertropical regions of Africa?

Cotton and karasia fields interrupted the park-like scenery of this district, in green patches of which wheat and onions were met with.

At Kano, in the twelfth parallel of latitude, among other caravans arriving, was one from Garja, with the desired kola-nut. Numbers of cattle are daily butchered on one side of the market-place. Kano is a great manufacturing town, especially in the weaving of cotton cloths, and the subsequent dyeing of them with indigo, which is cultivated in large quantities in this region. Barth tells us that milk, during the whole of his journey, formed his greatest luxury; but he adds the cautionary advice, that an African traveler should be particularly careful in the use of this article, which is capable of destroying a weak stomach entirely; and he would do better to make it a rule always to mix it with a little water, or to have it boiled. Better is the food which, on his journey to Kukawa, through Bornu, he received, as a "glorious lunch;" it consisted of a well-cooked paste of Negro millet with sour milk. On this route, which is east-northeast from Kano to Kukawa, a district was entered which may be called the exclusive region of the dum-palm or *Crucifera Thebaica*, the fruit of which we spoke of in our last notice. Before arriving at this place Barth received, at a village where he stopped, an immense bowl of milk, and a little fresh butter as cleanly prepared as in any English or Swiss dairy. This was prepared and brought to him by a Fellata,—for all over Bornu no butter is prepared but with the dirty and disgusting addition of some cow's urine, and it is all in a fluid state.

Shortly before reaching Kukawa, a peculiar kind of holcus called masakwa, (*Holcus Cornuus,*) was seen: it forms a very important article in the agriculture of Bornu. Sown after the end of the rainy season in shallow hollows, consisting of black sedimentary soil, it grows up entirely by the fructifying power of the latter, and ripens with the assistance only of the abundant dews, which fall here usually in the months following the rainy season. At Kukawa, as well as almost all over this part of Negroland, the great markets do not begin to be well attended till the heat of the day grows intense. In Yoruba, on the contrary, almost all the markets are held in the cool of the evening. In addition to the daily markets, there is held, every Monday, at Kukawa, a great fair, at which nearly 15,000 people congregate. Here are met with the inhabitants of all the eastern parts of Bornu, the Shuwa and the Keyam, with their corn and butter. The former, though of Arab origin, and still preserving in purity his ancient character, always carries his merchandise on the back of oxen, the women being mounted on the top of it; while the African Keyam employs the camel, if not exclusively, at least with a decided preference. The Kanembu, with their butter and dried fish, are very often seen in the market. All the luxuries offered to the people here are very few in comparison with the varieties of cakes and sweetmeats in the market-places of Hausa, or at Tasawa, Kaltena, and Kano, in the intermediate country. "Kolche," (the common sweet ground-nut,) "gangaola" (the bitter ground-hut,) boiled beans or "ngalo," and a few dry dates from the Tebu country, are almost the only things, besides water and a little nasty sour milk, offered as refreshment to the exhausted customer.

Barth, during his residence at Kukawa, made an excursion to Lake Tsad, or the Tsad, as it is generally called, ten miles distant, in an easterly direction. This led them over swampy plains in which he encountered large herds of "kelara," or antelopes, sometimes swimming, at others running. In a creek the only inhabitants found were the ngarutus, or river horses, which live here in great numbers, snorting about in every direction, with two species of crocodiles for company. At Maduwan, a village on the lake, Barth fared sumptuously, in having for supper fowls and a roasted sheep. In the villages along the shore of the lake are numerous herds of cattle, amounting altogether, it was computed, to eleven thousand head. Before reaching Káwa, the travelers passed over fields planted with cotton and beans. There they were feasted with a kind of "bolóbaló," or water mixed with pounded argum or dukhn, sour milk and meat.

On the route from Kukawa to Yola, which last place is in 9° 10′ N. lat., the travelers were obliged, on the first night, to be satisfied with cold "diggwa," a sweetmeat made of meal, honey, and butter. Farther on they entered a dense forest, where they gathered wild fruits, principally one called "foti," of the size of an apricot, and with three large kernels, the pulp of which was very pleasant. On the following morning they breakfasted upon "chebchebe," a light and palatable Kanuri sweetmeat, and upon "nufu," or habb'el aziz, dug up in large quantities almost over the whole of Bornu. On this route Barth saw again, but in greater abundance than before, the toso, the fruit of the *Bassia Parkii*, which consists almost entirely of a large kernel, of the color and size of a chestnut, which is covered with a thin pulp inside the green peel. This pulp has a very agreeable taste, but it is so thin that it is scarcely worth sucking out. A good deal of butter is prepared from the kernel, which is not only esteemed for seasoning the food of the people, but, also, for medicinal qualities ascribed to it. In the same region he speaks of the katakirri, a bulbous root, sometimes of the size of a large English potato, the pulp being not unlike that of the large radish, but softer, more succulent, and also very refreshing and nutritious. The juice has a milky color. A man, continues Dr. Barth, may easily travel a whole day with nothing to eat but this root, which seems to be very common during the rainy season, in the woody and moist districts of Central Africa. It is no less frequent near the Niger and in Kébbi, than it is here, but it is not seen in Bornu nor in Bmigari.

The *A'danseonia*, or baobab, (mon keybread-tree,) is the most common representative of the vegetable kingdom through the whole of Central

Africa. It is the constant follower of human society, spreading its gigantic branches out like an immense candelabrum. The negro can scarcely live without it; for how could he season his simple food without the baobab's young fresh leaves, or sweeten and flavor his drink without the slightly acid pulp, wherein the kernels are imbedded?

In the eastern quarter of Isege the travelers reached a fine sheet of water of considerable depth, stretching from east to west, and full of large fish, which seem to be of the same kind as that caught in the Tsad. Near the village of Kofa and the banks of a river, they passed by a forest, from which, among other fruits, they procured the toso, or rather its thin pulp, and the beautiful cream-fruit of the gonda-bush, (*Annona Palustris?*) their favorite dainties. Again they found a yellow fruit "gaudi," already referred to; it is of the size of an apricot, with a very thick peel, and, instead of a rich pulp, five large kernels filling almost the whole interior, but covered with a thin pulp of a very agreeable taste, something like the gonda. When speaking of the agreeable cool draughts obtained from the river, Barth adds his own observations to those of so many others on the same subject, in contrasting pure water with the unwholesome stagnant kind procured from pools, and impregnated with vegetable matter, and very often full of worms, so as to form certainly one of the chief causes of disease to the traveler. At the village of Lahaula, there is scarcely a single cow, but much vegetable butter is made; it has also a great deal of excellent honey. In his journeying on he tried the fruit of the deleb-palm, which was just ripe, (June 12th;) but it is not to be compared to the banana, and still less to the fruit of the gonda-tree. It is, however, of importance to the natives, and it yields, like the fruit of the dum-palm, a good seasoning for some of their simple dishes. This latter has an extensive range of growth, through nearly the whole breadth of Central Africa, from Kordofan to the Atlantic.

Ground-nuts form here a very large proportion of the food of the people, just in the same proportion as potatoes do in Europe, and the crops of corn having failed the last year, (1850,) the people had very little else besides. Nature, in these countries, as Barth well observes, has provided everything—dishes, bottles, and drinking-vessels (in the various *cucurbitaceæ*) are growing on the trees, rice in the forest, and the soil, without any labor, produces grain. Porridge is made from both kinds of the ground-nut, of which the more oily is represented to be the most wholesome. For making oil it is evidently the more valuable of the two. The Fulbe in this part of the country make also a similar porridge of sesamum. Different species of grain are cultivated in different soils. In Mbutude, millet, gero, or *Pennisteum typhoideum* was cultivated almost exclusively; here (in Adamawa) it is the dawa, "bain," (in Tulfulde) or sorghum, and principally the red sort, or "bairi boderi." The ground-nuts are cultivated between the corn, in the same way as beans are in many parts of Negroland. About Budanjo the land is extremely fertile, and besides sorghum or holcus, produced dankah or sweet potatoes, goya or yams, manive, and a great quantity of gunna, a large variety of calabash. At Melago the corn—*Pennisteum* or gero—stood already (June 17th) five feet high. At Chabajaule, the whole caravan fell to the charge of a single household, but, notwithstanding this, sufficient quantities, not only of "nyiro," the common dish of Indian-corn, but even of meat, were brought in the evening.

Rice is cultivated to a considerable extent in the western part of Adamawa. It grows, however, wild in many parts, from the southern provinces of Bornu, Bmigari, and Waday, as far north as El Haudh and Baghena, on the border of the western desert. The country of Tumbina is one of the finest of Central Africa, irrigated as it is by numerous rivers, among which the Benivwé and the Faro are the most important. The grain most commonly grown here, is *Holcus Sorghum*, but in this respect there is a great difference between the districts. Thus the country of the Mbum round Nygaundere scarcely produces anything but ruogo or yams, which form the daily and almost sole food of the inhabitants. Ground-nuts are plentiful both in the eastern and western districts. In the district of Tibati there is great exuberance of vegetation; the traveler sees both kinds of the banana, the gonda or popaya, several species of the giro-tree, the Pandanus, the *Kajilia*, the monkeybread-tree, and the *Banbanx*. Of the Palm tribe, the deleb-palm and the *Elais* Guineensis, (oil palm.) The date-tree is very rare. Among the bushes, the Palma Christi or *Ricinus* is extremely common. Altogether, the predominant tree in the southern provinces of Adamawa seems to be the banana.

On the journey from Kukawa to Kanem, round the north side of Lake Tsad, the travelers came to Yo, the principal food of the inhabitants of which is fish. Cotton and small quantities of wheat are the only produce of this region, besides fish and the fruit of the *Cucifera* or dum-palm, which forms an essential condiment for the "Kunu," a kind of soup made of Negro millet. Here Barth saw a specimen of the electric-fish about ten inches long, and very fat, which was able to benumb the arm of a man for several minutes. As he approached the Tsad the whole country began to be clothed with the sewak, or *Caparis Sodata*. Salt, as we had occasion to say before, is prepared from the ashes of the nuts of this plant: it is, also, procured from a peculiar species of grass growing in the river; and, by the Musgu, from the ashes of the stalks of millet and Indian-corn. In the vicinity of the lake an interesting and an uncommon sight was exhibited in a herd of elephants, no less than ninety-six in number. Turtle-soup made from animals of a small size (probably our terrapin,) was a luxury enjoyed by the travelers. They are most abundant round the town of Air, farther north. In Kanem the travelers accustomed themselves to camel's milk, and found it by degrees more palatable and wholesome than the milk of cows. Barth attributed his recovery from great exhaustion, the effect of fever, to this sort of diet.

Joining a warlike expedition from Kukawa to Mandara, the travelers came to Musa, the chief agricultural produce of which consisted of sweet sorghum, or *Sorghum Sacharatum*, regarded as a luxury by the Africans. Some of the stalks measured fourteen feet; but in the luxuriant valleys of the Kebbe they reach to twice this height! Pieces of the pith or marrow of this plant, which is of a snowy whiteness, was sent, as a dainty, by the vizier. No doubt this variety of the sorghum would yield sugar abundantly; but the sugar-cane itself grows wild in several parts of Negroland, and a small plantation of it, and boiling-houses on a small scale, were carried on by a native in the neighborhood of Sokoto. The deleb-palm, or fan-palm, probably only a variety of the Borassus flabelliformis, is in those regions the most common and predominant representative of the vegetable kingdom. From the Musgu country it seems to spread in an almost unbroken line through the southern provinces of Bmigari and Waday, as far as Kerdofam.

Two African luxuries, in the meat line, are unknown to the civilized palates, viz., the flesh of the giraffe and that of the elephant. The latter is very apt to derange weak bowels.

It is mentioned by Barth, in praise of onions, that, when cut into slices and mixed with tamarinds, they make a cool and refreshing drink.

In the territory of Logon hogs are found in immense numbers. Here, also, corn and tobacco are cultivated in friendly community. Water-melons are grown to some extent, also the squash-gourd, *Cucurbita Melopepo.* The *Croton Tiglium* is abundant in this region.

During his long stay at Bmigari, Barth subsisted almost entirely on a variety of grass, (*Poa Abyssenica,*) with the addition of a little rice. The Poa is very palatable when prepared with plenty of butter, or even boiled in milk.

We glean from the details just given as well as from those furnished in our last article, that, although the food of the great body of the inhabitants, both of Northern and Central Africa, is chiefly vegetable, yet that the dietary of those who can afford it, and especially in the regions of the last-mentioned division, is as various and implies as omnivorous a taste and digestion among them as among ourselves, or in the most favored parts of Europe. Dietetic lessons of this kind, furnished on a large scale, must be more instructive, as they ought to be more authoritative, than the minute and limited observations made on the gastronomic powers of persons leading an artificial and luxurious life.

